# Relationship between perceived social support and postpartum care attendance in three Latin American countries: a cross-sectional analytic study

**DOI:** 10.1186/s41256-021-00196-1

**Published:** 2021-05-07

**Authors:** Nancy R. Cardona Cordero, José Perez Ramos, Zahira Quiñones Tavarez, Scott McIntosh, Esteban Avendaño, Carmen DiMare, Deborah J. Ossip, Timothy De Ver Dye

**Affiliations:** 1 University of Rochester, School of Medicine and Dentistry, Obstetrics and Gynecology Department Research Division, 601 Elmwood Avenue, Rochester, NY 14642 USA; 2 Pontificia Universidad Católica Madre y Maestra, Santiago De Los Caballeros, 51000 República Dominicana; 3 Universidad de Ciencias Médicas, 400 metros oeste del M.A.G., Carr. Vieja a Escazú, San José, 10108 Costa Rica

**Keywords:** Postpartum care, Social support, Maternal health, Latin America, Costa Rica, Dominican Republic, Honduras, Social determinants

## Abstract

**Background:**

Postpartum Care is a strategy to improve survival of women and newborns, especially in low- and middle-income countries. Early post-partum care can promote healthy behaviors and the identification of risk factors associated with poorer pregnancy-related outcomes. The objective of this study was to assess the association of perceived social support with attendance to post-partum care in women from three Latin-American and Caribbean countries: Costa Rica, Dominican Republic and Honduras.

**Methods:**

Women aged 18+ who completed a pregnancy in the past 5 years were interviewed in local healthcare and community settings in each country. Perceived social support (PSS) was the primary explanatory variable and the primary outcome was self-reported attendance to post-partum care. Odds Ratios (OR) with 95% confidence intervals derived from logistic regression documented the association between variables. Adjusted Odds Ratios (AOR) were calculated, controlling for social and pregnancy-related confounders. Hosmer– Lemeshow’s Goodness-of-Fit statistic was computed to assess model fit.

**Results:**

Our cohort of 1199 women across the three Latin-American and Caribbean countries showed relatively high attendance to post-partum care (82.6%, *n* = 990). However, 51.7% (*n* = 581) of women reported lower levels of total PSS. Women were more likely to attend postpartum care if they had mean and higher levels of PSS Family subscale (OR: 1.9, 95%CI: 1.4, 2.7), Friends subscale (OR 1.3, 95%CI: 0.9,1.8), Significant Other subscale (OR 1.8, 95%CI: 1.3, 2.4) and the Total PSS (OR 1.8, 95%CI: 1.3, 2.5). All associations were statistically significant at *p* < 0.05, with exception of the Friends subscale. Women with higher levels of total PSS were more likely to attend to post-partum care (AOR:1.40, 0.97, 1.92) even after controlling for confounders (education, country, and food insecurity).

**Conclusions:**

Women with higher perceived social support levels were more likely to attend to post-partum care. From all countries, women from Dominican Republic had lower perceived social support levels and this may influence attendance at post-partum care for this subgroup. Societal and geographic factors can act as determinants when evaluating perceived social support during pregnancy.

**Supplementary Information:**

The online version contains supplementary material available at 10.1186/s41256-021-00196-1.

## Background

Women transitioning from the antepartum phase of pregnancy-related care to the postpartum phase face simultaneous challenges that include learning new maternal skills, making reproductive choices, and maintaining their personal and family health. Globally, almost 40% of women experience complications after delivery and 15% develop life-threatening problems [1]. Postpartum care (PPC) is a critical time that provides an opportunity to assess and enhance women’s physical, social, and psychological health. In low- and middle-income countries, PPC has been a interventional tool to decrease maternal and neonatal morbidity and mortality [1, 2].

An essential component of PPC is the postpartum visit, and as many as 40% of women do not attend follow-up postpartum [3]. In 2014, the World Health Organization recommended that a woman and her child should receive postpartum care 24 h after delivery and at least three more times. In low income countries, however, between 20 and 35% of women use any PPC services [2]. Low use of postpartum services is associated with lack of education, poverty, and limited access to health care [4]. In fact, the poorest 20% of the population in most Latin American and Caribbean (LAC) countries is still lagging behind toward reducing inequalities in reproductive and maternal-child health [5]. Likewise, reproductive and maternal interventions are more inequitable than those delivered to children in LAC [5]. Therefore, attendance to care and risks during the postpartum period may affect populations disproportionally and renewed actions are needed to improve maternal health equity.

Studies have also shown that adaptive maternal behaviors can be influenced by a woman’s perceptions of the amount of positive support she is receiving [6]. Greater social support has been linked to better health status and as a mediator for stress during pregnancy [7] and the postpartum period. Social support has been defined as ‘*an exchange of resources between at least two individuals perceived by the provider or recipient to be intended to enhance the well-being of the recipient.*’ [8] Perceived social support is an indicator of social support adequacy (e.g., network structure and received support) when compared to the needs and expectations of the individual [9]. Therefore, social support can be defined by structure depending on the relationship with its recipient (family, friends, significant others, professionals) or function (informational, instrumental, emotional, appraisal) [10]. Further, social support may impact a woman’s adaptation and transition process and influence her immediate decisions and promote postpartum maternal health.

Social determinants in countries undergoing a rapid health transition such as LAC countries, may affect disproportionally sub-populations in maternal, reproductive, and child health. When evaluating the national composite coverage index stratified by wealth in LAC countries, Costa Rica, Honduras and the Dominican Republic have a similar absolute inequality between the groups. Additionally, evidence suggests that up to 2010 infant mortality has fallen and life expectancy has increased gradually for these countries, but there is no information about the influence that postpartum care may have in maternal health. Prior research has focused on how information and communication technologies (ICT) have influenced maternal health in selected LAC countries [11]. The aim of this study was to determine attendance to postpartum care in areas of three LAC countries: Costa Rica, Honduras and Dominican Republic and to evaluate its relationship with perceived social support and associated factors. Results can serve as a baseline for the scientific community and practitioners where interventions can be implemented and maternal health can be improved for LAC.

## Methods

### Setting and sample

This cross-sectional study was nested within a larger NIH-funded maternal health research and training initiative (*MundoComm*; www.mundocomm.org) and evaluated differences in postpartum care attendance, its relationship with perceived social support, and affiliated social, demographic, and pregnancy-related characteristics. To assess these behaviors, a survey was developed and administered to women of rural communities in Costa Rica, Dominican Republic, and Honduras.

### Survey development

The survey was divided into topics including socio-demographics, maternal health, access to information and communication technologies, use of social media, antepartum and postpartum care. Relevant to the current analyses, the survey included an adaptation of validated psychosocial scales including the Multidimensional Scale of Perceived Social Support (PSS) [12]. The survey was developed in English and translated into Spanish, then back-translated into English using the Brislin method [13]. Several iterations of the survey were pretested individually by each in-country team [14]. Feedback during these processes helped to adapt the survey to become culturally- and linguistically- relevant for the participating countries.

### Measures

Sociodemographic characteristics were assessed from the first section of the survey and transformed into categorical values. Binary categories were created for maternal education, marital status and maternal age, which was based on the mean age for our cohort. Worries about enough food during pregnancy was a four-option question and was transformed into two food insecure categories. Country of residence included three categories (Costa Rica, Dominican Republic, and Honduras). Characteristics of pregnancy were acquired from different sections of the survey and transformed into categorical values. Intentions of becoming pregnant in the woman’s most recent completed pregnancy were assessed using the CDC Pregnancy Risk Assessment Monitoring System (PRAMS)‘s standardized question: “Thinking back to just before you got pregnant with your new baby, how did you feel about becoming pregnant?” [15]. Additional questions included were based on the CDC’s Reproductive Health Survey series [16]. Questions include: *1) Before your last pregnancy, were you trying to become pregnant? 2) how many children have you have that live with you and how many children do you have that don’t live with you? 3) did you try breastfeed? 4) does your baby came before expected time? 5) did you seek postpartum care on your last pregnancy?* Complications during the first 6 weeks of delivery were also assessed and included severe bleeding, bad vaginal smell, infected surgery wounds, fainting, comma, high fever, pain urination, pelvic pain, breast infection, continuous urinary leak, and fecal flow. We used CDC’s Health-Related Quality of Life 14 (HRQOL-14) General Health Measure to assess self-described health status [17]. Additionally, a question asked if a health services provider had told the participant that they had a chronic health condition. Both variables were transformed into dichotomous categorical variables. The Multidimensional Score of Perceived Social Support [12] was assessed and divided into three standardized subscales (Family, Friends, Significant Other) and a Total Scale (four items for each sub-score and 12 for the total score). PSS was analyzed several ways: 1) as a continuous variable (standardized T-score (mean = 50; standard deviation = 10) [18] and 2) as a dichotomous categorical variable cut at the mean of the raw PSS score, low (8.4 or less) and high (Over 8.4).

### Data collection and processing

Study participants were women age 18 years and older, had at least one pregnancy in the 5 years prior to the study (regardless of outcome), and had to be able to read and verbally consent to participate. A house-to-house approach and snowball sampling methodology was used to recruit participants. Recruitment occurred during the Spring and Summer of 2017 in the province of Heredia in Costa Rica, the department of Olancho in Honduras, and in the province of Santiago in the Dominican Republic.

### Data analysis

All statistical analyses were performed using IBM SPSS statistical software, Version 25 (IBM SPSS Statistics for Windows, Version 27.0. Armonk, NY: IBM Corp). The independent variables in the analysis were country of residence, maternal age, education, marital status, food insecurity, perception of health, health problems, becoming pregnant, first time mother, and premature baby. The dependent variable in these analyses was postpartum care attendance. We used descriptive statistics to examine the relationship and describe population characteristics (n and %). The main explanatory variable was total perceived social support score and respective subscales. We tested the distribution of continuous variables (PSS Total Score and subscales) using the Normality test. PSS values for each subscale and total score were transformed to z-scores and then into standardized t-scores for comparison [18]. The correlation coefficient was calculated for all sub-scales and the PSS Total score was used as the independent variable for further analyses. Analysis of variance (ANOVA) was used to test for differences between participants who attended postpartum care and those who did not by PSS subscale and PSS total score. Correlation between PSS subscales and total score was estimated using Spearman’s rank order correlation coefficient (See Supplemental material). Based on these results, we used PSS Total score as the main explanatory variable using high and low (higher than the mean and lower than the mean) categories. To assess the bivariate associations between attendance to postpartum care and PSS total score, we used bivariate logistic regression model. We computed crude Odds Ratios (OR) and 95% confidence intervals for potential confounders for both primary variables (PPC and PSS) considering demographic, social, health- and pregnancy related variables. We included potential confounders of primary relationship between PPC and PSS in our multivariate model in their *p*-value in both bivariate analyses was < 0.20 [19]. Potential confounding variables included sociodemographic (age, country of residence, education and food security), health (health problems, perception of health) and pregnancy-related (intended pregnancy, first-time mothers) characteristics. We used forward stepwise selection to add possible confounders and calculated adjusted OR (AOR). Hosmer – Lemeshow’s Goodness-of-Fit [19] statistic was computed to assess model fit. Statistical significance for quantitative analyses was determined if *p* < 0.05 (two-tailed tests).

### Regulatory process and ethical considerations

Multiple iterations of regulatory documents were assessed and addressed by each institutional review board. The MundoComm project was approved by the Universidad de Ciencias Médicas (UCIMED) FWA Institutional Review Board in Costa Rica and (with local approval) in Honduras (as a proxy, since Honduras did not have an FWA Institutional Review Board). In addition, in the Dominican Republic, the study was reviewed and approved by Consejo Nacional de Bioética en Salud (CONABIOS) at the national level and the Pontificia Universidad Católica Madre y Maestra (PUCMM)‘s FWA Institutional Review Board at the university level. Regulatory language for each country agency was incorporated in the survey. Additionally, the project was approved by the University of Rochester’s Research Subjects Review Board. All data collectors had completed appropriate ethics certification in their country.

## Results

### Sample characteristics

Our cohort consisted of 1199 women across countries (Costa Rica: 401; Dominican Republic: 404; Honduras: 394). Women’s age at time of interview was 27.1 years. Women were surveyed, on average, 27 months post-partum (with Honduran women interviewed significantly more recently at 23 months post-partum), and interviews lasted an average of 19 min, with no significant variation by country (data not shown). Demographic and pregnancy-related characteristics of our cohort are presented in Table 1. The majority of women were married or living as married 75% (*n* = 898) and less than 10% (*n* = 107) had attained an educational level higher than high school. Although most women (*n* = 822, 68%) perceived their health status as good, very good or excellent, 23% (*n* = 285) reported to have at least one health-related problem. In our study population, 8% (*n* = 101) of postpartum women to reported have gestational diabetes and 24% (*n* = 274) reported hypertension. Pregnancy was desired by 497 (41%) of the women, and 311 (26%) of the women were first-time mothers. In total, 14% (*n* = 171) of women had delivered at fewer than 37 weeks of pregnancy.

**Table 1:** Women’s socio-demographic and pregnancy-related characteristics

Demographics & Health	N	%	Pregnancy-related	N	%
Country	1199				
Costa Rica	401	33.44	Becoming Pregnant now	1186	
Dominican Republic	404	33.69	Yes	497	41.91
Honduras	394	32.86	No	689	58.09
Age	1197		First time mother	1177	
18–27 yrs	669	44.11	Yes	311	26.42
28+ yrs	528	55.89	No	866	73.58
School	1161		Gestational diabetes	1195	
High school or less	1044	89.92	Yes	101	8.45
More than Highschool	107	9.22	No	1094	91.55
Married or living as married	1192		Hypertension during pregnancy	1135	
Yes	898	75.34	Yes	274	24.14
No	294	24.66	No	855	75.33
Health Problems	1199		Food insecure during pregnancy	1197	
At least one	285	23.77	Yes (One or more times)	549	45.86
None	914	76.23	No	648	54.14
Perception of Health	1196		Premature baby	1194	
Good	822	68.73	Yes	171	14.32
Bad	374	31.27	No	1023	85.68

Overall, 82% (*n* = 990) of women attended to PPC and the majority implemented healthy PPC habits such as breastfeeding (*n* = 1126, 94%) (Table 2). In most women (*n* = 787, 81%), postpartum care occurred 10 days or less which is consistent with the perception of 83% (*n* = 801) of women who agreed that attendance should happen in less than 10 days of partum. Women opted for first-level centers (*n* = 526, 53%) as their main location to receive postpartum care, followed by public (*n* = 256, 25%) and private (*n* = 188, 19%) hospitals. Postpartum care services were mostly provided by doctors or doctor’s assistants (75%), followed by nurses. At 6 weeks of postpartum, the most common complications reported were pelvic pain (*n* = 366, 30%), urination pain (*n* = 235, 19%), and severe bleeding (*n* = 185, 15%).

**Table 2:** Women’s Postpartum-related characteristics

Postpartum-related	N	%	Complications of partum	N	%
Attended Postpartum Care	1199		Severe bleeding	1195	
Yes	990	82.60	Yes	185	15.48
No	209	17.40	No	1010	84.52
Place where received postpartum care	989		Very bad smell	1193	
Public hospital	256	25.88	Yes	63	5.28
Private hospital	188	19.01	No	1130	94.72
First level center	526	53.19	Infect surgery wounds	1194	
My house	1	0.10	Yes	94	7.87
Other place	15	1.52	No	1100	92.13
Dispensary	3	0.30	Fainting	1192	
			Yes	98	8.22
Who provided postpartum care service	1121		No	1094	91.78
Doctor or doctor assistant	843	75.20	Coma	1185	
Clinical assistant	10	0.89	Yes	2	0.17
Nurse	205	18.29	No	1183	99.83
Midwife	4	0.36	High fever	1194	
Pediatric helper	27	2.41	Yes	147	12.31
Public health worker	19	1.69	No	1057	88.53
Other	13	1.16	Painful urination	1190	
Days attended postpartum care	965		Yes	235	19.75
0–3 days	213	22.07	No	955	80.25
4–6 days	193	20.00	Pelvic pain	1195	
7–10 days	381	39.48	Yes	366	30.63
> 10 days	178	18.45	No	829	69.37
Days should attend postpartum care	957		Breast infection	1191	
0–3 days	232	24.24	Yes	64	5.37
4–6 days	207	21.63	No	1127	94.63
7–10 days	362	37.83	Continuous urinary leak	1189	
> 10 days	156	16.30	Yes	52	4.37
			No	1137	95.63
Breastfeed	1195		Fecal flow	1188	
Yes	1126	94.23	Yes	3	0.25
No	69	5.77	No	1185	99.75

### Main findings

Perceived Social Support (PSS) was evaluated as a standardized scale and as a categorical variable (low vs high) and results are shown in Table 3. Standardized median scores for our cohort varied by subcategory as follows: Standardized PSS scores indicate that women who attended PPC had slightly higher mean levels in all PSS subscales when compared to their counterparts; comparison between and within these groups was statistically significant (*p* < 0.02) (Table 3). When transforming PSS to a categorical variable, Friends PSS subscale was the only subscale where the majority of women (55.6%, *n* = 654) had higher PSS. Nonetheless, PPC attendance was strongly statistically associated (*p* < 0.001) with higher levels of PSS by subscale when evaluating the bivariate relationship. In fact, women were more likely to attend postpartum care if they had mean and higher levels of PSS Family subscale (OR: 1.9, 95%CI: 1.4–2.7), Significant Other subscale (OR 1.8,95%CI: 1.3–2.4) and Total score (OR 1.8, 95%CI:1.3–2.5) (Table 4).

**Table 3:** Perceived Social Support by subscale and comparison with postpartum care attendance

Categories and measures		Perceived Social Support Subscales			
		Family	Friends	Significant Other	Total
Total	n	1190	1177	1190	1134
Perceived Social Support Standardized T-score	Median	46.6	52.3	46.6	49.9
Perceived Social Support Categorical values	Low - n (%)	675 (57.6)	523 (44.4)	613 (51.5)	586 (51.8)
	High - n (%)	496 (42.4)	654 (55.6)	577 (47.5)	546 (48.2)
Received Postpartum Care	n	974	964	974	925
	Mean (SD)	50.6 (9.8)	50.3 (10.1)	50.6 (9.8)	50.6 (9.9)
	Confidence Interval	49.9–51.2	49.6–50.9	49.9–51.2	49.9–51.3
Did not receive Postpartum Care	n	206	203	206	199
	Mean (SD)	47.1 (10.5)	48.4 (9.5)	47.1 (10.5)	47.1 (9.9)
	Confidence Interval	45.7–48.6	47.12–49.8	45.7–48.6	45.7–48.5
Mean Square	Between groups	1990.5	583.0	1990.5	1971.6
	Within groups	98.6	99.6	98.6	98.5
ANOVA	p	0.001	0.016	0.001	0.001

**Table 4:** Bivariate relationship between Postpartum Care and Perceived Social Support by subscale

Women centered variables		Postpartum Care n (%)		OR	95% C.I.		Sig.	
	N	Yes	No		Lower	Upper		
PSS Friends	1167							
Lower than the mean	520	103 (50.7)	417 (43.3)	Referent				
Mean and Higher	647	100 (49.3)	547 (56.7)	1.4	1.0	1.8	0.052	
PSS Significant Other	1180							
Lower than the mean	607	131 (63.6)	476 (48.9)	Referent				
Mean and Higher	573	75 (36.4)	498 (51.1)	1.8	1.3	2.5	0.001	*
PSS Family	1161							
Lower than the mean	668	145 (70.4)	523 (54.8)	Referent				
Mean and Higher	493	61 (29.6)	432 (45.2)	2.0	1.4	2.7	0.001	*
PSS Total	1124							
Lower than the mean	581	127 (63.8)	454 (49.1)	Referent				
Mean and Higher	543	72 (36.2)	471 (50.9)	1.8	1.3	2.5	0.001	*

* *p* < 0.05

Bivariate analyses for both primary variables (PPC and PSS) indicated that country of residence, age group, level of education, perception of health, health problems, intentions of pregnancy, and first-time pregnancy could potentially act as confounders (see supplemental material). In brief, postpartum care attendance was higher among women who intended their pregnancy, first-time mothers, and providing breastfeeding. As well, experiencing other health and pregnancy related complications such as high fever was also significantly associated with postpartum care attendance in our study. Meanwhile, health conditions developed during pregnancy (gestational diabetes and hypertension) were evaluated in this study, but no significance differences were observed.

Unadjusted and adjusted models assessed the relationship between post-partum care and perceived social support, controlling for potential confounders (Table 5). The first model represents the unadjusted relationship between the PCC and Total PSS. The second model added country of residence; the third model added food insecurity, and the fourth model added educational level. The odds of attendance to postpartum care changed from 83% in the unadjusted relationship to 47% in the first multivariate model (Model 2). When controlling for two confounders (Model 3), the odds of attending postpartum care only varied 0.01%. Odds of attending postpartum care were adjusted to 40% for women who had higher levels of PSS, when adding the third confounder (Model 4). In all models’ higher levels of Total PSS remained statistically associated with PPC attendance (*p* < 0.05). Goodness-of-Fit statistics for all multivariate models were non-significant, indicating that the data fit the analytic models used. Women with higher levels of PSS were between 40 and 50% more likely to attend PPC, when controlling for confounders.

**Table 5:** Multivariate Models for Postpartum Care and Perceived Social Support

	OR	95% CI		***p***-value
**Model 1. Unadjusted**				
Higher Perceived Social Support	1.8	1.3	2.5	0.001
Lower Perceived Social Support	(Reference)			
**Model 2. Adjusted for Country**				
Higher Perceived Social Support	1.5	1.1	2.1	0.023
Lower Perceived Social Support	(Reference)			
**Model 3. Adjusted for Country and Food Insecurity**				
Higher Perceived Social Support	1.5	1.1	2.1	0.022
Lower Perceived Social Support	(Reference)			
**Model.4 Adjusted for Country, Food Insecurity and Education**				
Higher Perceived Social Support	1.4	1.0	1.9	0.049
Lower Perceived Social Support	(Reference)			

## Discussion

Latin American women’s perception of social support in this study was significantly associated with post-partum care participation. PPC and PSS differ, however, by country of residence, educational level, and social factors, such as food insecurity, and further studies should use interactions and stratified analyses to further understand these confounders and related implications. PPC is a critical time that provides an opportunity to assess and enhance women’s physical, social, and psychological health.

### Effect of health-related factors

Postpartum women may experience pain, discomfort, and disturbances in patterns associated with physical and mental health that include changes in their body appearance, nutrition, and sleep [20, 21]. These dramatic changes, adjustments, and difficulties are prompted by new demands, structural constraints, and other events in the postpartum period that can impact a woman’s psychosocial wellbeing. Wiegers et al. (2006) [21] revealed that first-time parents expressed the importance of postpartum care and its role in relation to baby-care and self-care information that is provided. The relationship between breastfeeding and postpartum care in our study has also been documented in other studies reporting a strong influence of postpartum care into breastfeeding, specifically after the first 2 weeks of birth [22]. Consistent with findings in our study, maternal behavior and women’s desire for more information on self-care and infant care is influenced by their perception of positive social support received [23]. Considering the social support perceived and related actions by postpartum mothers in LAC countries in our study may serve as indicative of the need for further evaluation of psychological and social factors that may change or influence actions during this vulnerable period. Another study [24] showed postpartum women linking attendance to care as an important resource for monitoring both, physical, and mental health.

Recent findings have shown that women with health conditions perceived postpartum care as essential to monitoring their health and as an intervention to prevent further complications [24]. A slightly higher attendance to postpartum care was observed in women who reported at least one health problem diagnosed by a health provider such as asthma, heart problems, cancer or high blood pressure, when compared to their counterparts. These results indicate that multidisciplinary approaches are fundamental to improve maternal health promotion strategies within health departments and health professional across LAC countries.

### Effect of societal-related factors

Poor health outcomes have been associated with low educational level in several studies including quality of live and maternal health studies [25]. Specifically, in postpartum health, low educational levels have been associated with low mood in the first postpartum week [26]. Although women with higher educational attainment were more likely to attend postpartum care when compared with woman who had lower educational level in our study, the vast majority of postpartum women had a lower educational level. Concerns emerging from these results include the possibility that educational level could act as an additional predictor affecting maternal health and a possible alternative towards healthy behaviors, the refocus of educational postpartum policies is crucial.

Maternal health studies have also evaluated social factors such as food insecurity and its role during pregnancy and in the postpartum period. A strong association has been reported between food insufficiency and postpartum depression [27]. Another study revealed that this association was higher in women that have lower levels of social support [28], but the presence of instrumental social support buffers food insufficiency. Consistent results were shown in our study, women who were food insecure also exhibit lower levels of perceived social support and food insecurity decreased the probability of attending postpartum care in our study.

Our findings also suggest that postpartum care attendance may vary by country of residence. Hondurans and Costa Ricans had higher odds of postpartum care attendance when compared with Dominicans. Likewise, women from the Dominican Republic exhibit lower levels of perceived social support when compared to their counterparts and this may influence attendance to post-partum care for this subgroup. While this difference was found in our study, studies evaluating social support experiences in communities in Dominican Republic [29] and in Costa Rica [30], women consistently reported lower levels of social support compared to men.

These inconsistent social support and postpartum health results between countries provide insights and a baseline for approaches targeting cultural and geographically perceptions and norms. Promoting healthy pregnancies, children, and women involves a partnership that includes critical-thinking, communication, and self-assessment, with individually-targeted objectives and collective strategies between and within countries. Tangible forms of material support, such as childcare and access to transportation, should be further evaluated given that they can be common barriers to seek care and enable postpartum visit attendance.

### Strengths and limitations

The parent study used a community-based, mixed-method approach with a sample size powered to detect the differences explored in this study. The survey was validated in every community to ensure the quality of the data collected. Maternal health inferences apply only to those communities and cannot be extrapolated to the population of each country. Nevertheless, these findings common to the three countries suggest that if other communities are sampled, the results might be similar. The healthcare system and access to healthcare in each country is different, and this difference could impact attendance to PPC.

## Conclusion

Social support has a direct impact on maternal role and satisfaction [7]. Attendance to postpartum care is crucial for both - a woman and her infant - and other internal and external factors can play a role. Findings in this study support that to fully understand social influences on women’s health during the postpartum period, we need to examine characteristics using a holistic approach from the individual- to the macro system level. Moving forward in closing these gaps, future studies and approaches could include community level interventions to address perceptions and qualitative studies to understand knowledge, attitudes, beliefs and reasons associated with social support. Additionally, given that social media use played a role mediating the effect of child death in LAC countries, the contribution of these elements should be evaluated.

## Supplementary Information

**Additional file 1.**

## Data Availability

The datasets used and/or analyzed during the current study are available from the corresponding author on reasonable request.
