# Supplementary material for: Relationship between perceived social support and postpartum care attendance in three Latin American countries: a cross-sectional analytic study
Source: Glob Health Res Policy. 2021 May 7;6:16. doi: 10.1186/s41256-021-00196-1 (PMC8103641; doi:10.1186/s41256-021-00196-1)
Supplement: Supplementary file 1 — Additional file 1. [file 41256_2021_196_MOESM1_ESM.docx]

**Supplemental Table: Bivariate analyses of women-centered variables, postpartum care receipt, and total Perceived Social Support score**

| **Women centered variables** | | **Postpartum Care n (%)** | | **OR** | **95% C.I.** | | **Sig.** | |  | **Total Perceived Social Support** | | **OR** | **95% C.I.** | | **Sig.** | |
| --- | --- | --- | --- | --- | --- | --- | --- | --- | --- | --- | --- | --- | --- | --- | --- | --- |
|  | **N** | **Yes** | **No** |  | **Lower** | **Upper** |  |  | **N** | **High** | **Low** |  | **Lower** | **Upper** |  |  |
| **PSS Total** | 1124 | 925 (82.3) | 199 (17.7) |  |  |  |  |  | 1124 | 543 (48.3) | 581 (51.7) |  |  |  |  |  |
| Mean and Higher | 543 | 471 (86.7) | 72 (13.3) | 1.8 | 1.3 | 2.5 | 0.01 | * | 925 | 471 (50.9) | 454 (49.1) | 1.83 | 1.3 | 2.5 | 0 | * |
| Lower than the mean | 581 | 454 (78.1) | 127 (21.9) | Reference |  |  |  |  | 199 | 72 (36.2) | 127 (63.8) | Reference |  |  |  |  |
| **Country** | 1199 | 990 (82.6) | 209 (17.4) |  |  |  |  |  | 1134 | 547 (48.2) | 587 (51.8) |  |  |  |  |  |
| Costa Rica | 401 | 343(85.5) | 58 (14.5) | 2.4 | 1.7 | 3.4 | 0.01 | * | 384 | 221 (57.6) | 163 (42.4) | 2.8 | 2.1 | 3.8 | 0 | * |
| Honduras | 394 | 359 (91.1) | 35 (8.9) | 4.1 | 2.8 | 6.2 | 0.01 | * | 354 | 198 (55.9) | 156 (44.1) | 2.6 | 1.9 | 3.5 | 0 | * |
| Dominican Republic | 404 | 288 (71.3) | 116 (28.7) | Reference |  |  |  |  | 396 | 128 (32.3) | 268 (67.7) | Reference |  |  |  |  |
| **Age** | 1197 | 988 (82.7) | 209 (17.5) |  |  |  |  |  | 1132 | 546 (48.2) | 586 (51.8) |  |  |  |  |  |
| 28 - Highest | 527 | 447 (84.8) | 79 (15.2) | 1.3 | 1.0 | 1.8 | 0.06 | ** | 492 | 260 (53.0) | 230 (47.0) | 1.4 | 1.1 | 1.7 | 0 | * |
| Lowest-27 | 669 | 540 (80.7) | 129 (19.3) | Reference |  |  |  |  | 640 | 285 (44.5) | 355 (55.5) | Reference |  |  |  |  |
| **Education** | 1161 | 956 (82.3) | 205 (17.7) |  |  |  |  |  | 1101 | 534 (48.5) | 567 (51.5) |  |  |  |  |  |
| More than High School | 117 | 107 (91.5) | 10 (8.5) | 2.5 | 1.3 | 4.8 | 0.01 | * | 110 | 75 (68.2) | 35 (31.8) | 2.5 | 1.6 | 3.8 | 0 | * |
| High School or less | 1045 | 850 (81.3) | 196 (18.7) | Reference |  |  |  |  | 991 | 459 (46.3) | 532 (53.7) | Reference |  |  |  |  |
| **Married or living as married** | 1197 | 984 (82.6) | 208 (17.4) |  |  |  |  |  | 1127 | 546 (48.4) | 581 (51.6) |  |  |  |  |  |
| Yes | 898 | 737 (82.1) | 161 (17.9) | 0.9 | 0.6 | 1.2 | 0.45 |  | 849 | 416 (49.0) | 433 (51.0) | 1.1 | 0.83 | 1.4 | 0.5 |  |
| No | 294 | 247 (84.0) | 47 (16.0) | Reference |  |  |  |  | 278 | 130 (46.8) | 148 (53.2) | Reference |  |  |  |  |
| **Health problems** | 1199 | 990 (82.6) | 209 (17.4) |  |  |  |  |  | 1134 | 547 (48.2) | 587 (51.8) |  |  |  |  |  |
| None | 913 | 756 (82.8) | 156 (17.2) | 1.1 | 0.7 | 1.5 | 0.68 |  | 275 | 119 (43.3) | 156 (56.7) | 1.3 | 0.9 | 1.7 | 0.1 | ** |
| At least one | 285 | 233 (81.8) | 52 (18.2) | Reference |  |  |  |  | 859 | 428 (49.8) | 431 (50.2) | Reference |  |  |  |  |
| **Food Insecurity** | 1199 | 989 (82.6) | 208 (17.4) |  |  |  |  |  | 1132 | 547 (48.3) | 585 (51.7) |  |  |  |  |  |
| Yes (One or more times) | 549 | 439 (80.0) | 110 (20.0) | 0.7 | 0.5 | 1.0 | 0.02 | * | 519 | 252 (48.6) | 267 (51.4) | 1.0 | 0.7 | 1.2 | 0.9 |  |
| None | 648 | 550 (84.9) | 98 (15.1) | Reference |  |  |  |  | 613 | 295 (48.1) | 318 (51.9) | Reference |  |  |  |  |
| **Perception of good health** | 1196 | 987 (82.5) | 209 (17.5) |  |  |  |  |  | 1132 | 547 (48.3) | 585 (51.7) |  |  |  |  |  |
| Yes | 822 | 685 (83.3) | 137 (16.7) | 1.2 | 0.9 | 1.7 | 0.27 |  | 777 | 404 (52.0) | 373 (48.0) | 1.6 | 1.2 | 2.1 | 0 | * |
| No | 374 | 302 (80.7) | 72 (19.3) | Reference |  |  |  |  | 355 | 143 (40.3) | 212 (59.7) | Reference |  |  |  |  |
| **Intended pregnancy** | 1186 | 977 (82.4) | 209 (17.6) |  |  |  |  |  | 1119 | 536 (47.9) | 583 (52.1) |  |  |  |  |  |
| Yes | 497 | 421 (84.7) | 76 (15.3) | 1.3 | 1.0 | 1.8 | 0.07 | ** | 469 | 237 (50.5) | 232 (49.5) | 1.2 | 0.9 | 1.5 | 0.1 | ** |
| No | 689 | 556 (80.7) | 133 (19.3) | Reference |  |  |  |  | 650 | 299 (46.0) | 351 (54.0) | Reference |  |  |  |  |
| **Premature baby** | 1194 | 985 (82.5) | 209 (17.5) |  |  |  |  |  | 1128 | 544 (48.2) | 584 (51.8) |  |  |  |  |  |
| Yes | 171 | 139 (81.3) | 32 (18.7) | 0.9 | 0.6 | 1.4 | 0.65 |  | 161 | 77 (47.8) | 84 (52.2) | 0.98 | 0.7 | 1.3 | 0.9 |  |
| No | 1023 | 846 (82.7) | 177 (17.3) | Reference |  |  |  |  | 967 | 467 (48.3) | 500 (51.7) | Reference |  |  |  |  |
| **Breastfeed** | 1195 | 988 (82.7) | 207 (17.3) |  |  |  |  |  | 1128 | 545 (48.3) | 583 (51.7) |  |  |  |  |  |
| Yes | 1126 | 935 (83.0) | 191 (17.0) | 1.5 | 0.8 | 2.6 | 0.19 |  | 1062 | 510 (48.0) | 552 (52.0) | 0.8 | 0.5 | 1.3 | 0.4 |  |
| No | 69 | 53 (76.8) | 16 (23.2) | Reference |  |  |  |  | 66 | 35 (53.0) | 31 (47.0) | Reference |  |  |  |  |
| **First time mothers** | 1177 | 969 (82.3) | 208 (17.7) |  |  |  |  |  | 1114 | 537 (48.2) | 577 (51.8) |  |  |  |  |  |
| Yes | 311 | 263 (84.6) | 48 (15.4) | 1.2 | 0.9 | 1.8 | 0.23 |  | 292 | 152 (52.1) | 140 (47.9) | 1.2 | 0.9 | 1.6 | 0.1 | ** |
| No | 866 | 706 (81.5) | 160 (18.5) | Reference |  |  |  |  | 822 | 385 (46.8) | 437 (53.2) | Reference |  |  |  |  |
| * p < 0.05,** p < 0.20 | |  |  |  |  |  |  |  |  |  |  |  |  |  |  |  |
|  |  |  |  |  |  |  |  |  |  |  |  |  |  |  |  |  |
